# Microbiota and Metabolite Profiling Reveal Specific Alterations in Bacterial Community Structure and Environment in the Cystic Fibrosis Airway during Exacerbation

**DOI:** 10.1371/journal.pone.0082432

**Published:** 2013-12-17

**Authors:** Kate B. Twomey, Mark Alston, Shi-Qi An, Oisin J. O'Connell, Yvonne McCarthy, David Swarbreck, Melanie Febrer, J. Maxwell Dow, Barry J. Plant, Robert P. Ryan

**Affiliations:** 1 Department of Microbiology, Biosciences Institute, University College Cork, Cork, Ireland; 2 The Genome Analysis Centre, Norwich Research Park, Norwich, United Kingdom; 3 Division of Molecular Microbiology, College of Life Sciences, University of Dundee, Dundee, United Kingdom; 4 Cork Adult Cystic Fibrosis Centre, Department of Respiratory Medicine, Cork University Hospital, University College Cork, Cork, Ireland; 5 Genomic Sequencing Unit, Division of Molecular Medicine, Colleges of Life Sciences, University of Dundee, Dundee, United Kingdom; University Medical Center Utrecht, The Netherlands

## Abstract

Chronic polymicrobial infections of the lung are the foremost cause of morbidity and mortality in cystic fibrosis (CF) patients. The composition of the microbial flora of the airway alters considerably during infection, particularly during patient exacerbation. An understanding of which organisms are growing, their environment and their behaviour in the airway is of importance for designing antibiotic treatment regimes and for patient prognosis. To this end, we have analysed sputum samples taken from separate cohorts of CF and non-CF subjects for metabolites and in parallel, and we have examined both isolated DNA and RNA for the presence of 16S rRNA genes and transcripts by high-throughput sequencing of amplicon or cDNA libraries. This analysis revealed that although the population size of all dominant orders of bacteria as measured by DNA- and RNA- based methods are similar, greater discrepancies are seen with less prevalent organisms, some of which we associated with CF for the first time. Additionally, we identified a strong relationship between the abundance of specific anaerobes and fluctuations in several metabolites including lactate and putrescine during patient exacerbation. This study has hence identified organisms whose occurrence within the CF microbiome has been hitherto unreported and has revealed potential metabolic biomarkers for exacerbation.

## Introduction

Cystic fibrosis (CF) is the most common lethal autosomal recessively inherited disorder of Europids. It is caused by a mutation in the Cystic Fibrosis Transmembrane Conductance Regulator (*CFTR*) gene that makes those diagnosed extremely susceptible to pulmonary infections. This is in part due to overproduction of mucus in the airways that predisposes patients to microbial colonisation, which is the major cause of morbidity and mortality. Although a limited number of bacterial species including *Pseudomonas aeruginosa, Burkholderia cenocepacia, Staphylococcus aureus* and *Haemophilus influenzae* have been established as important CF pathogens [1], it is now appreciated that CF airway infections are more broadly polymicrobial in nature. Furthermore, culture-independent methodologies have revealed that the bacterial communities present are even more diverse than previously realised [2], [3]. A range of these techniques has been deployed: generation of 16S rRNA clone libraries [4], [5], terminal restriction fragment length polymorphism analysis [2], [6], microarray hybridisation [7], phylochip analysis [8] and pyrosequencing [9]. Collectively these studies demonstrate that a number of previously unrecognised aerobic and anaerobic species are present in CF airways, some of which represent new potential pathogens.

The strategies to uncover bacterial diversity described above rely on using total DNA extracted directly from the sample of interest and as a consequence are unable to discern between DNA from metabolically active, latent or dead bacteria. This is a considerable drawback for the selection of antimicrobial therapies to be deployed in the treatment of CF lung infection as these should be targeted towards those populations of bacteria that are metabolically active, and hence sensitive to the agents [2], [10], [11]. Furthermore, microbes are subjected to numerous selective pressures and nutritional or other environmental cues that can control bacterial behaviour and response to therapy [12]–[14]. Unfortunately information concerning the metabolic activity of microbes in the CF airway during infection is limited. To date, metabolomic methods have been applied to bronchoalveolar lavage fluid, model CF cell culture systems and to the examination of regulatory lipid mediators in adult CF sputum [15]–[17]. Determining the relative abundance of metabolically active bacteria and the metabolite composition during CF infections may be imperative in the design of diagnosis strategies, tailoring prescription of antibiotics and informing treatment regimes. To address this issue, we have used a next-generation sequencing approach to evaluate the diversity of total and metabolically active bacteria associated with the CF airway. We isolated both DNA and RNA from sputum samples taken from two separate cohorts of CF patients and non-CF subjects for analysis of 16S rRNA genes and transcripts. Concurrently a metabolic fingerprinting approach was applied to the sputum samples to acquire an insight of the low-molecular-weight molecules present.

## Results

### Collection of sputum samples from adult CF and non-CF patients

To explore the relationship between respiratory tract bacterial community and metabolite composition of CF lung disease, we began by enrolling 80 patients of a long-term program at the Adult CF clinic at Cork University Hospital. The patients recruited for this cross-sectional study included 75 CF patients and 5 non-CF patients with stable bronchiectasis. The average age of the patient population was 28.3 years [range: 19–52]. The patient group consisted of 44% females. Further clinical description and analysis of these patients recruited is reported in Table S1. During enrolment we collected 110 lower airway expectorated samples: 75 samples were collected during a period of stability, 26 during a period of acute exacerbation and 9 sputum samples from non-CF patients around the same period as defined in the Material and Methods and by Fuchs *et al.*, [18]. Henceforth, these three cohorts of samples are referred to as “stable” and “exacerbated” and “control” samples, respectively.

### Diversity and abundance of total and active bacteria from CF airway

To determine the composition of the bacterial community in each sputum sample from the stable, exacerbated and control cohorts, we assessed 16S rRNA profiles amplified from total DNA and reverse-transcribed RNA extracted from each cohort sample. This entailed amplifying 16S rRNA genes from 110 samples (in duplicate) using specific primers that spanned variable regions V3 to V5 and incorporating specific bar code tags for identification. Amplicon libraries were prepared and sequenced using Roche 454 FLX titanium technology, as described in the Materials and Methods and reported previously [19]. The analysis detected a total of 60,000 PCR amplicons that were ∼350 bp in length, with, on average, >1500 reads for each sample (Table S2). Using a 98% similarity threshold value, we identified a wide range of operational taxonomic units (OTUs) for each sample. On average, 400 OTUs per sample were detected. After normalisation to the sample with the smallest number of reads (≈5000), 401 OTUs were included in further analyses. The number of sequences from each genus/order was used to determine the predominant bacteria that were present. A complete summary of the read assignments for each patient cohort is provided in Table S2.

#### Dominant bacteria

**Figure 1:**
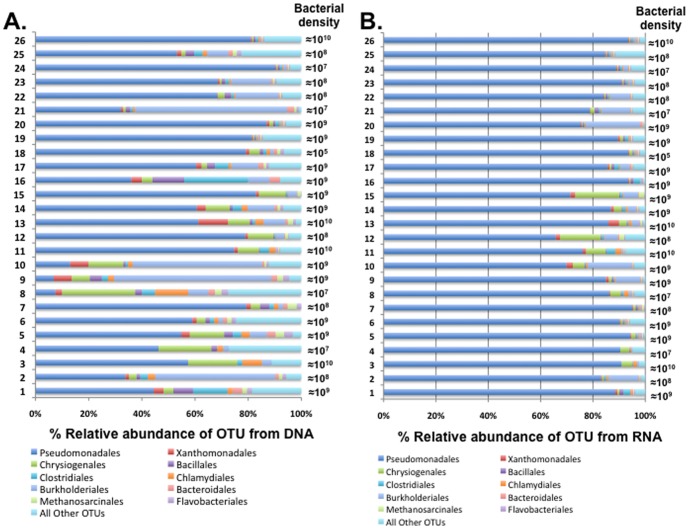
Relative abundances of bacterial orders identified as operational taxonomic units (OTUs) from the sequence reads generated from sputum samples of CF patients presenting with exacerbation. The coloured segments of each bar represent the proportion of reads mapping to different bacterial orders. Percentage of sequences from total DNA (A) or total transcribed RNA (B) are described. Details of clinical parameters and microbiological assessment of each of the sputum samples collected are described in Table S3. Total bacterial density in each sputum sample examined is indicated as 16S rRNA copies/ml sputum as measured by quantitative PCR.

The 10 most abundant orders of bacteria observed by DNA-based analysis in stable and exacerbated cohorts were *Pseudomonadales*, *Xanthomonadales*, *Chrysiogenales*, *Bacillales*, *Clostridiales*, *Chlamydiales*, *Burkholderiales*, *Bacteroidales*, *Methanosarcinales* and *Flavobacteriales* (FIG. 1 and FIG. S1). Reassuringly, the predominant aerobic pathogens (*Pseudomonas* and *Burkholderia*) detected by culture were recognised by sequencing to occur in the corresponding patient samples (Table S3). The identification of these orders in both stable and exacerbated patients is consistent with recently reported findings [8], [9]. These ten orders accounted for 89% of the reads detected from the stable cohort but a greater proportion of the exacerbated cohort (96%). *Pseudomonadales, among* which the major contributor was *P. aeruginosa*, was the dominant order in all subjects in the exacerbated cohort, demonstrating a reduction in bacterial richness in exacerbated patients consistent with previous reports (recently reviewed in [20]).

Not unexpectedly, the principal bacterial orders found in the control cohort have been previously identified as normal flora of the upper respiratory airway and oropharynx including *Propionibacterium*, *Corynebacterium*, *Staphylococcus spp.*, *Neisseria*, *Haemophilus*, and anaerobic lineages such as *Prevotella*, *Veillonella*, and *Fusobacterium spp.* (data not shown).

The 16S rRNA gene data set generated from genomic DNA represents the total community including dormant or dead bacteria. In contrast, the 16S rRNA data set generated from reverse-transcribed RNA indicates the community of metabolically active bacteria, i.e. those with higher ribosomal content. Comparison of these data sets showed that the ten most abundant bacterial orders as revealed by analysis of reverse-transcribed RNA were identical to and had the same relative abundance as those identified by the DNA-based methods in both stable and exacerbated cohorts (FIG. 1; FIG. S1; FIG. S2). Furthermore, the bacterial community revealed by DNA analysis did not contain members that were absent in the community described by the 16S rRNA data set. However, the DNA assessment appears to overestimate the abundance of less prevalent organisms (including anaerobes) to different degrees such that it skews the assessment of the relative abundance of these bacterial orders (FIG. 1; FIG. S1; FIG. S2). Notably, quantitative PCR analysis revealed no significant differences in total bacterial densities in sputum samples from stable and exacerbated cohorts (FIG. 1; FIG. S1).

#### Anaerobes

**Figure 2:**
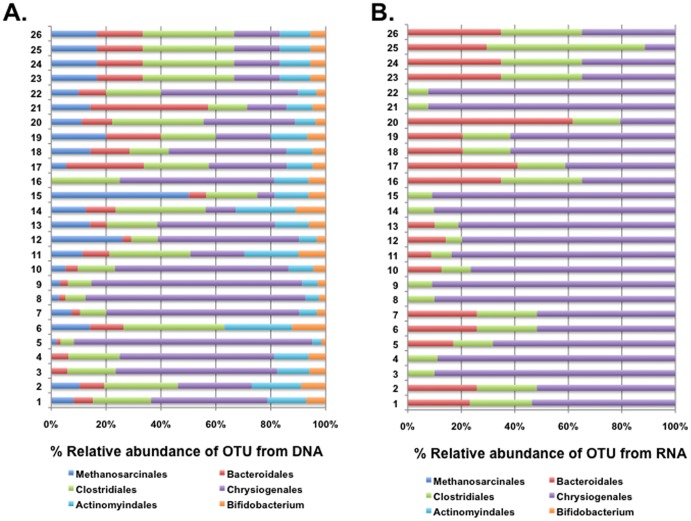
Identification of anaerobic bacterial populations among sputum samples from CF patients presenting with exacerbation. Percentage sequences from DNA-based (A) and RNA-based (B) approaches are described. The coloured segments of each bar represent the proportion of reads mapping to different anaerobic bacterial orders.

In the current study, only strict anaerobes were classed as anaerobes, whereas, facultative anaerobes were classed as aerobes. In agreement with previous studies [21], [22], we also found strictly anaerobic bacteria to be diverse and abundant within the CF airways. Strict anaerobes accounted for between 1–2% of the reads detected in both the stable and exacerbated cohorts. The abundance of strict anaerobes *in total* did not appear to alter between total and metabolically active populations in both the stable and exacerbated cohorts (FIG. 2; FIG. S3). The abundant orders in both stable and exacerbated cohorts were *Methanosarcinales*, *Bacteroidales*, *Clostridiales*, *Chrysiogenales*, *Actinomyindales* and *Bifidobacteriales*. The complexity of the anaerobe community was considerably reduced in samples from the exacerbated cohort as revealed by both DNA-based and RNA-based methods. The RNA-based analysis suggested that the anaerobe community in exacerbated patients was comprised almost entirely of *Bacteroidales*, *Clostridiales* and *Chrysiogenales*, with the last being the predominant order in the majority of cases (FIG. 2). In contrast, DNA analysis detected, in addition, bacteria of the orders *Methanosarcinales*, *Actinomyindales* and *Bifidobacteriales*.The few bacteria that have been classified within the order *Chrysiogenales* are environmental organisms able to respire arsenate or selenium [23], [24]. As far as we are aware however, no association of bacteria from this order with CF or other infections has been made previously.

#### Other significant orders of bacteria

**Figure 3:**
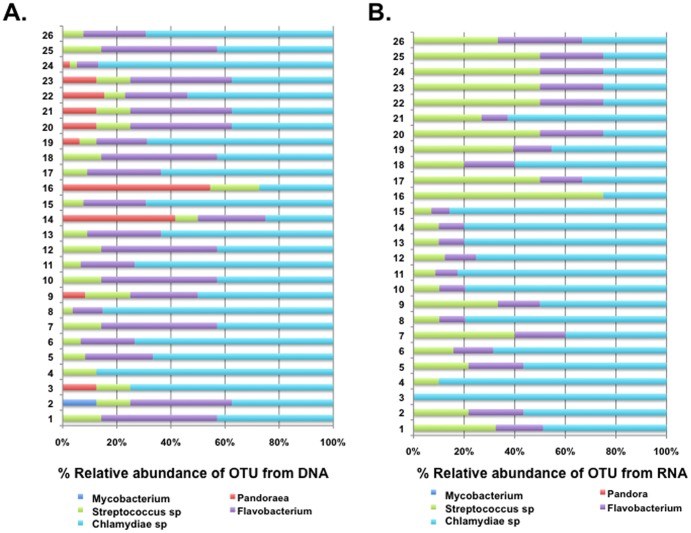
Prevalence of emerging CF bacterial pathogens among sputum samples from CF patients suffering exacerbation. Percentage of sequences from DNA-based (A) or RNA-based (B) approaches are delineated. The coloured segments of each bar represent the proportion of reads mapping to different bacterial orders.

Numerous other aerobic bacteria are believed to contribute to the community complexity of the CF lung disease [9]. We examined the prevalence of various genera identified by previous reports to be important [9]. The genera examined included *Mycobacterium* sp., *Streptococcus sp*., *Pandoraea* sp., *Rothia* sp., *Flavobacterium* sp., and *Chlamydia sp.* (FIG. 3; FIG. S4). These genera found within most samples comprised <3% of the total amplicon population. RNA-based analysis did not detect *Pandoraea* sp. or *Mycobacterium* sp. in the exacerbated cohort (FIG. 3).

### Community structure as revealed by DNA-based (total) and RNA-based (metabolically-active) techniques

**Figure 4:**
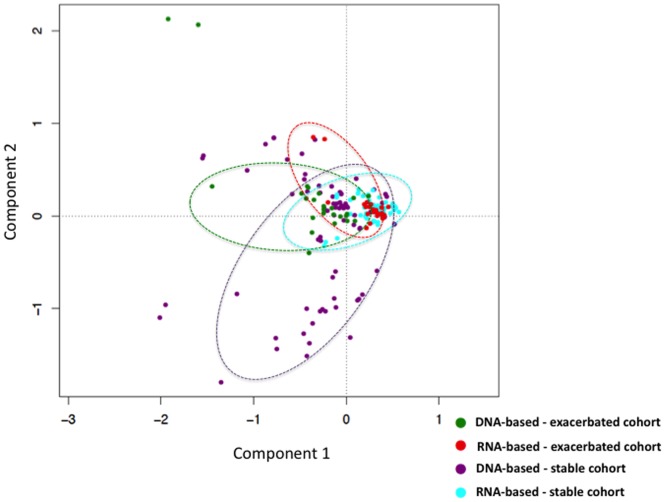
Bacterial community structures of samples from stable CF patients and those presenting with exacerbation as plotted based on Bray–Curtis (BC) distance metric. Each community from each sample is represented as a filled circle and coloured by class (green – total DNA extracted from CF patients presenting with exacerbation; red – total RNA extracted from CF patients presenting with exacerbation; magenta – total DNA extracted from stable CF patients; cyan – total RNA extracted from stable CF patients). BC sample clusters are based on the distribution of the bacterial orders found in an individual sample. A confirmation of differences between samples was validated using the ade4 permutation test. This determined the statistical significance of the BC; the ellipses represent the 95% confidence region.

We compared community structures across patient cohorts to determine if inter-individual differences in structure exceeded the changes we observed between samples. Pairwise ecologic distances were calculated for all samples using the Bray–Curtis (BC) distance metric, which takes into account both community membership and relative abundance. These distances then were visualised by comparing stable and exacerbated cohorts revealing appreciably distinct communities (FIG. 4). Bacterial communities revealed by DNA-based and RNA-based techniques in stable cohorts appear sporadically distributed with only a slight overlap at the centre of the ordination diagram. The majority of the metabolically-active (RNA-based) populations from stable patients clustered around this point. Interestingly, with the exacerbated cohort considerable overlap and clustering between communities from DNA-based and RNA-based datasets was seen, with only a small number of outliers. Together, these findings reflect that in single sample community analysis using either DNA- or RNA-based techniques, extensive variability is seen in communities identified in stable samples, whereas little variability is seen in bacterial communities from exacerbated samples, where in the majority of cases, *Pseudomonas aeruginosa* is the predominant organism.

### Standard culture profiling of CF airway

In parallel to the above studies, sputum samples were screened using standard CF microbiology culture-based protocols as detailed in the Methods section. Members of four phyla were identified with the majority of isolates belonging to the gamma subdivision of the Proteobacteria (Table S3). As expected, mucoid and non-mucoid *Pseudomonas*, *Staphylococcus aureus*, and bacteria from the genera *Burkholderia* and *Haemophilus* dominate the microbiology profile. Other organisms such as *Streptococcus* and *Mycobacterium* spp. were less commonly found, but no members of the oropharyngeal flora were detected. Comparison of culture-based profiling and detection by RNA-based analysis of sputum showed that both methods detect dominant CF pathogens, but the RNA-based method provides a broader perspective of the CF airway microbiome. This is consistent with previous studies [20].

### Metabolite profiling of the secretions of the CF airways

**Figure 5:**
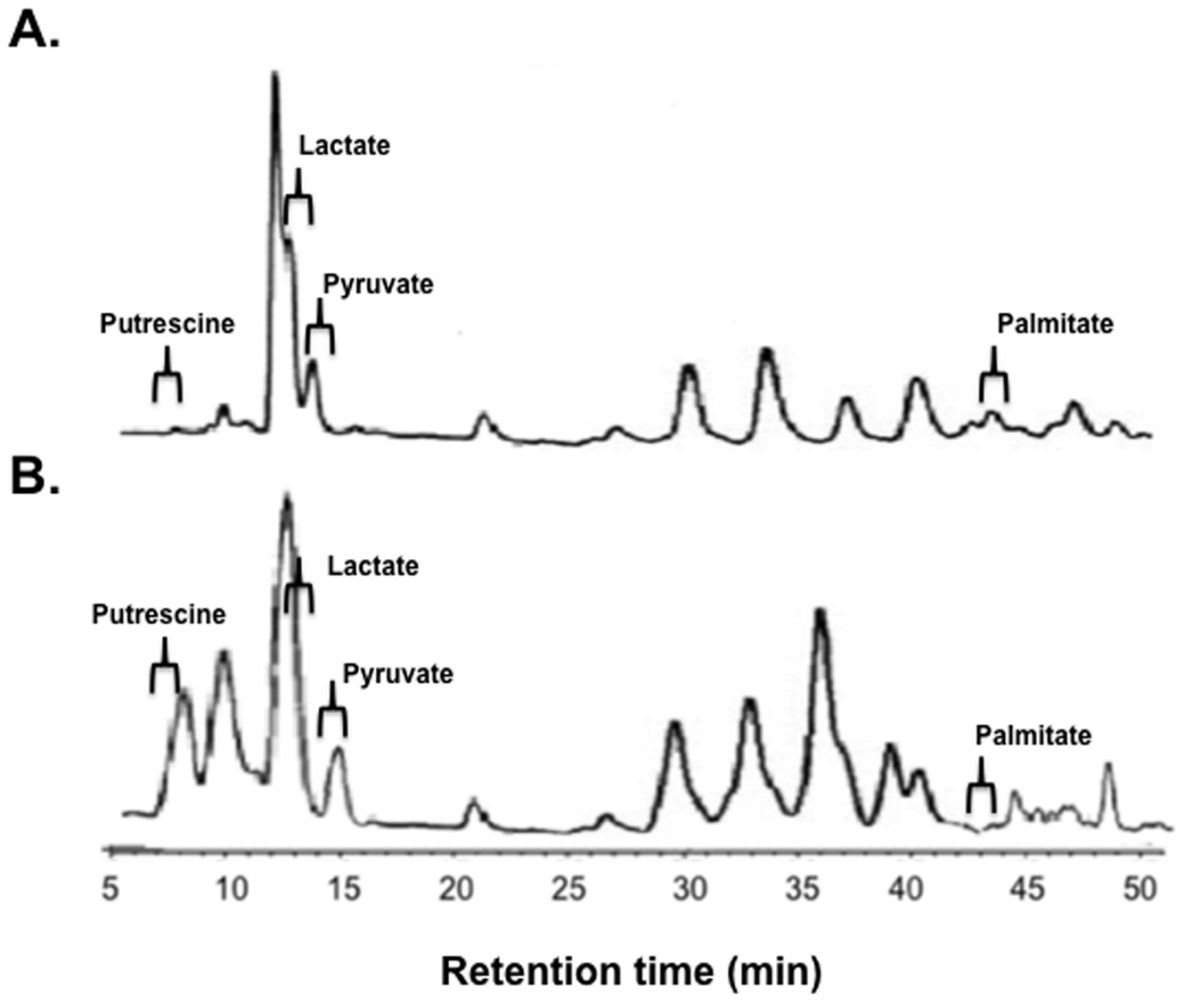
Representative HPLC trace of metabolites from sputum taken from a CF patient during a period of stability (A) and presenting with pulmonary exacerbation (B). Regions are labelled to indicate a range were metabolites of interest that were recovered from samples. Specific concentrations of metabolites identified are detailed in Tables S4 and S5.

Metabolite profiling of the sputum samples was carried out using an LC-MS/MS approach. Samples were taken from 26 CF patients during a period of stability and also during exacerbation. The microbiome in these sputum samples was also profiled. From each of these sputum samples, 200 small molecules were detected, of which 47 could be identified and 36 (listed in Table S4) could be quantified. These compounds included amino acids, polyamines, and carbohydrates. A representative HPLC profile is presented in FIG. 5.

**Figure 6:**
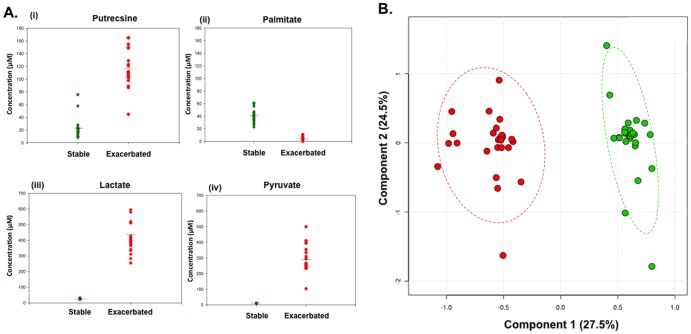
Concentrations of lactate (i), pyruvate (ii), putrescine (iii) and palmitate (iv) detected in sputum samples taken from 26 CF patients during a period of stability and presenting with pulmonary exacerbation (A). Each dot represents an individual patient. Data are given as the average values measured in triplicate (metabolite concentrations are described as µM unless otherwise stated and means ± standard deviations are reported). (B) PLS-DA plots of metabolite profile of sputum taken from 26 CF patients during a period of stability and presenting with pulmonary exacerbation. Stable subjects (green) vs. exacerbated subjects (red); R^2^ = 0.95, Q^2^ = 0.93 for a two-component model. The ellipses represent the 95% confidence region. Permutation tests (n = 1,000) validated the model (p<0.001).

The differences in the metabolite levels between stable and exacerbated samples and their statistical significance were calculated (see Materials and methods). Multi-variate analysis was then employed to identify metabolites that showed reproducible changes between stable and exacerbated cohort across the three replicates, with p<0.001 deemed to be significant. Of the 36 metabolites that were quantitated, 32 showed no statistically significant variation in level between all of the samples. However, statistically significant fluctuations were clearly seen in levels of pyruvate, lactate, palmitate, and putrescine (FIG. 6A; FIG. S5). Specifically, pyruvate, lactate and putrescine showed significant increases in samples from exacerbated compared to stable patients, whereas palmitate levels decreased. In summary, we detected significant changes in the level of a small number of metabolites associated with sputum samples between stable and exacerbated patients.

### Correlation between the levels of altered metabolites and the composition of the bacterial community

Previous studies have shown that metabolite compositions in clinical scenarios such as Crohn's disease and urogenital infection can be correlated with microbiota composition and host physiology [25], [26]. This prompted us to examine any possible relationship between the composition of the respiratory tract bacterial community and the metabolite profile of CF sputum. To do this, we merged the data sets describing the levels of metabolites and the RNA-based composition of the bacterial community for each patient. Initial PCA analysis showed a trend for separation according to clinical status (stable or exacerbated). Differences in metabolites between stable and exacerbated were highly correlated with changes in the total CF microbiota and provided evidence of the microbial diversity having an impact on the small molecules found in sputum (FIG. 6B). We further statistically characterized the abundance of specific metabolites for a relationship to the identified dominant CF microbiota. An assessment of the total population identified a relationship between the increase in putrescine, pyruvate and lactate and the abundance of *Pseudomonas* and the strict anaerobes of the order *Chrysiogenales*. The correlation with the level of *Pseudomonas* is consistent with previous reports that *P. aeruginosa* dominates the microbiome during exacerbation. Interestingly however, the correlation between the level of these metabolites and the level of *Chrysiogenales* is stronger (p≤0.012) than that with *Pseudomonas* (p≤0.05).

## Discussion

In this study, we have applied the parallel technologies of ribosomal tag sequencing based on DNA and RNA and metabolomics to sputum samples taken from separate cohorts of CF and non-CF subjects as an approach to characterise the ‘active’ bacterial community and environment in the CF airway. This analysis revealed (i) that significant differences in particular metabolites occur in sputum taken from clinically stable CF patients and those presenting with CF pulmonary exacerbation, (ii) that although the population size of all dominant orders of bacteria as measured by DNA- and RNA-based methods are close, greater discrepancies are seen with less prevalent organisms, some of which we associate with CF for the first time, and (iii) that a strong relationship exists between the abundance of specific strict anaerobes and fluctuations in the level of several metabolites during CF pulmonary exacerbation.

### Alteration in metabolites of the CF airway during exacerbation

We have shown that sputum from patients presenting with a CF pulmonary exacerbation have elevated levels in pyruvate, lactate, and putrescine compared with sputum taken from clinically stable CF patients or non-CF patients. Conversely, levels of palmitate were considerably higher in clinically stable CF patients than those levels observed in sputum samples taken from CF patients with exacerbation. Both host and microbial cells could contribute to these changes in metabolite profile. Elevated levels of the anaerobic glycolysis metabolites pyruvate and lactate in CF respiratory secretions have already been associated with increased hypoxic conditions during CF exacerbation [27]. Under anaerobic growth conditions in the absence of alternative electron acceptors, *P. aeruginosa* utilises the conversion of pyruvate into acetate and lactate for long-term survival [28]. Recent work has shown that pyruvate fermentation is required for microcolony (biofilm) formation by *P. aeruginosa* [29], which is consonant with an earlier suggestion that pyruvate fermentation might contribute to the survival of *P. aeruginosa* in biofilms during infection [28].

A recent study examining the contribution of arginase activity to airway nitric oxide deficiency in CF demonstrated that levels of various polyamines, including spermine, were significantly increased in sputum taken from CF patients with exacerbation [30]. Interestingly, polyamines have been previously identified in the outer membrane and attached to lipopolysaccharide of Gram-negative bacteria, including *P. aeruginosa* and have been recently linked to surface-associated functions that serve to protect the bacterial outer membrane from oxidative stress and the action of antibiotics [31]. Such effects could promote bacterial colonisation and proliferation during CF airway exacerbation. To our knowledge, a decrease in palmitate levels associated with a CF pulmonary exacerbation has not been previously reported. This may be of importance since airway surfactant components, particularly lipids, are essential for proper airway function and a lack of such components leads to respiratory distress. *P. aeruginosa* has been shown to adapt to utilise abundant lipid molecules when growing under hypoxic conditions, which might account partially for this reduction in this specific lipid [32]. It is unlikely that the microorganisms colonising the CF airway are the sole contributors to the alteration in levels of specific metabolites in CF sputa, which reflects the complex interplay between human and bacterial processes. Nevertheless, if longitudinal studies could establish that the changes identified preceded onset of exacerbation as measured by clinical parameters, they may provide potential biomarkers that would provide an early report of the need for intervention.

### Alteration in the anaerobic bacterial population during CF exacerbation

In this present study, we mapped the bacterial community structure during periods of stability and exacerbation in the CF airway using both DNA and RNA-based approaches. It was clear from our study that the two approaches gave different estimates of the relative abundance of less widespread organisms. This may have considerable implications in measuring antibiotic impact and subsequent treatment success of CF airway infections [11]. Both methods have limitations; DNA-based approaches undoubtedly overestimate the abundance of various bacterial orders since they do not differentiate between bacteria that are metabolically active and those that are metabolically inactive (or dead). However, determination of relative abundance based on RNA-based methods makes the assumption that all living bacteria are equally metabolically active, which may not necessarily be the case [33].

This approach also allowed us to identify the *Chrysiogenales* as being the predominant order of anaerobic organism in CF patients undergoing exacerbation. To our knowledge this is the first description that bacteria of the order *Chrysiogenales* are associated with CF lung disease. The absence of previous reports of *Chrysiogenales* in CF may reflect difficulties in their cultivation. The only member of this order that has been characterised thus far is *Chrysiogenes arsenatis*, an obligately anaerobic organism isolated from estuarine environments that respires arsenate or selenium [23], [24]. Selected typed strains of *Chrysiogenes arsenatis* are at various stages of genome sequencing [34] and a draft genome sequence is available. Isolation of specific CF isolates and genome sequencing would be of considerable benefit in advancing our limited understanding of Chrysiogenales and their possible contribution to disease and to promotion of pulmonary exacerbations. Such effects may be exerted through interactions with the host or through microbe-microbe interactions with more prevalent pathogens, as has been seen for organisms such as *Stenotrophomonas maltophilia* that have the ability to influence the behaviour of *P. aeruginosa* [35], [36].

In conclusion, the work in this paper adds to a number of reports that suggest the significance of less prevalent organisms (to include strict anaerobes) in CF infection, which may have implications for therapeutic regimes [21], [37].

## Materials and Methods

### Enrolment of subjects, ethics statement and sputum sample collection

Sputum samples from 80 CF patients (January 2010–September 2010) were collected from the Adult Cystic Fibrosis Treatment centre in the Cork University Hospital. All study participants provided written consent. The Clinical Research Ethics Committee of the Cork Teaching Hospitals (CREC) granted full approval to the project. Sputum samples were assigned into two clinical states; stable and exacerbated based on medical history, which was assessed by physician observations, patient-reported symptoms on the day of collection and antibiotic use within 30 days before collection. Sputa from non-CF patients with bronchiectasis were also collected for control analysis. All sputa were analysed, then frozen at −80°C in 1 ml aliquots for further study. Patient clinical parameters were also recorded including the participant's age, lung function, CFTR genotype, pulmonary administered antibiotics, and colonisation by microorganisms identified by standard clinical laboratory cultivation techniques. Solid media included Chocolate-anaerobic agar (CHOC), MacConkey agar (MAC), Polymyxin B- MacConkey agar, Mannitol-salt agar (MSA), Colisitin-nalidixic acid agar. Plates were incubated at 37°C in the presence of 5% CO_2_ for two days with the exception of OFPBL cultures, which were incubated at 30°C and chocolate agar cultures which were incubated anaerobically.

### Definition of pulmonary exacerbation

Pulmonary exacerbations in CF patients have been defined as the clinical need for additional treatment as indicated by a change in clinical parameters as described by Fuchs *et al.*, [18]. Significant pulmonary symptoms include (but are not restricted to) increase in cough, change in sputum production (volume and/or appearance), onset or increase of haemoptysis, weight loss, exhaustion and fatigue and/or lung function decline. Any combination of these symptoms has been considered sufficient for additional treatment.

### Nucleic acid extraction

Prior to DNA extraction, sputum samples were washed in sodium phosphate buffer to remove adherent saliva. DNA and RNA extraction from sputum samples was carried out as previously described [19]. All reagents, glassware and plastics used in RNA work were DEPC-treated prior to use. RNA was extracted as follows: 0.75 ml of Tri Reagent (Sigma-Aldrich) was added to approximately 0.2 ml of each sample and vortexed for 1 minute. Samples were incubated at room temperature for 5 minutes prior to the addition of 0.2 ml chloroform. Samples were vortexed for 15 seconds and incubated at room temperature for 5 minutes. Phases were separated by centrifugation at 12,000×*g* for 15 minutes at 4°C. For isolation of DNA 0.3 ml of 100% ethanol was added to precipitate the DNA from the lower phase. The sample was mixed by inversion, incubated at room temperature for 3 minutes and centrifuged at 12,000×*g* for 5 minutes at 4°C. The pellet was washed in 0.1 M sodium citrate, 10% ethanol solution (during each wash the pellet was allowed to stand for at least 30 minutes). Pellets were centrifuged at 12,000×*g* for 5 minutes at 4°C and washed twice in 75% ethanol. The DNA was vacuum dried, with the pellet re-suspended in 50 µl H_2_O and stored at −20°C. For isolation of RNA the upper phase was transferred to a fresh microfuge tube and 0.5 ml of propan-2-ol was added. Samples were incubated for 10 minutes at room temperature and RNA was pelleted by centrifugation at 12,000×*g* for 10 minutes at 4°C. The supernatant was removed and the RNA pellet washed once in 75% ethanol and re-pelleted by centrifugation at 7,500 ×*g* for 5 min at 4°C. Pellets were air-dried for 10 minutes, re-suspended in 30 µl distilled water and incubated for 10 minutes at 55°C. Purified RNA samples were stored as aliquots at −70°C. Prior to reverse transcription, any residual DNA was removed using DNaseI (Epicentre) in accordance with the manufacturer's instructions, with PCR amplification controls performed as appropriate.

### Quantitative reverse transcriptase PCR

Total bacterial density was determined using a Taqman® Universal PCR Mastermix assay, in which a fragment of the 16S ribosomal RNA gene was amplified, as described previously [38]. Details of the relevant primers are available upon request. Total bacterial primers at a concentration of 100 nM each, and 1 µl of undiluted sputum DNA or cDNA were used in each reaction. The cycling conditions used are previously described [38]. Each run contained non-template control and a 10-fold dilution series of *P. aeruginosa* genomic DNA. All quantitative PCR analyses were performed in triplicate. Standard curve samples were converted from DNA concentration to 16S rRNA copy number based on four rRNA operon copies and 6, 264, 403 bp per *P. aeruginosa* PAO1 genome and used to extrapolate total 16S rRNA operon copy number from cycle threshold (CT) values from specimens samples.

### 16S rRNA universal PCR amplication and Roche 454 sequencing

The following universal 16S rRNA primers were used for the PCR reaction: KTF (5′-CCAGACTCCTACGGGAGGCAGC-3′) and KTR (5′-CCGTCAATTCCTTTGAGTTT-3′) for the V3–V5 region. Barcode sequences for the V3–V5 samples of either AGCAGAGC or AGCAGATG were attached between the 454 adaptor sequence and the forward primers. Standard PCR reaction conditions were employed for reactions with Taq polymerase −2 mM MgCl_2_, 200 nM each primer, 200 µM dNTPs. The PCR conditions established were 94°C for 50 seconds (initialisation and denaturing) followed by 40°C for 30 seconds (annealing), 72°C for 60 seconds in 40 cycles (extension), and a final elongation step at 72°C for 5 minutes. Two negative control reactions containing all components, but water instead of template, were performed alongside all test reactions, and were routinely free of PCR product, demonstrating lack of contamination with post-PCR product. The optimal annealing temperature for the primers, which included 454 adapters and barcode sequences, was empirically determined by gradient PCR using control reactions with initially purified bacterial genomic DNA, and validated on *Pseudomonas* gDNA. The 16S rRNA V3–V5 amplicons were subsequently sequenced on a Roche 454 Genome Sequencer FLX platform (The Genome Analysis Centre, UK) according to 454 protocols.

#### Sequence analysis and phylogenetic classification

Sequences (FASTA format) and their respective quality scores were obtained from SFF data files via Roche's 454 Sequencing System Software (v. 2.5.3). A file giving the forward primer sequence and defining samples by their barcodes was created to enable analysis by the mothur software package (v. 1.20.0; http://www.mothur.org/). Mismatches to the barcode (1 nt) and primer (2 nt) were allowed, but sequences were discarded from the analysis if: they contained any ambiguous bases, were of length <100 nt, or if the average quality score fell below 35 over a 50 nt window. Sequences were aligned using the SILVA alignment database (http://www.arb-silva.de/) and clustered for OTU analyses via the mothur analysis pipeline. Further sequence analysis such as clustering, characterisation of chimeras and sequence alignment was carried out as previously described [8], [9], [19]. The sequences reported in this paper were deposited in the Sequence Research Archive (SRA) at NCBI under the following accession numbers: ERA259841; ERX330942; ERX330943; ERX330944; ERX330945; ERX330946; ERX330947; ERX330948; ERX330949.

### Metabolomic profiling

The metabolomic platforms were described previously in detail [39]. Briefly, the platform consisted of two independent platforms: liquid chromatography/tandem mass spectrometry (LC/MS/MS) optimised for basic species, LC/MS/MS optimised for acidic species. The major components of the process are summarised as follows:

#### Sample Extraction and MS Analysis

Triplicate samples were extracted using an automated Hamilton liquid handling workstation in 400 µl of methanol, containing the recovery standards. The samples destined for MS analysis were dried under vacuum desiccation for a minimum of 24 hours and then derivatised under dried nitrogen using bistrimethyl-silyl-trifluoroacetamide. The GC column was 5% phenyl, and the temperature ramp was from 40 to 300°C in a 16-minute period. Samples were analysed on a Thermo-Finnigan Trace DSQ fast-scanning single-quadrupole mass spectrometer using electron impact ionization.

#### LC/MS/MS Analysis

Fractions were analysed by LC–MS–MS. Analyses were performed using a Shimadzu SCL-10a *VP* HPLC system connected directly to a Finnigan TSQ 7000 triple-quadrupole mass spectrometer via an electrospray interface. Two separate HPLC/MS injections were performed on each sample: one optimised for positive ions and one for negative ions. Chromatographic separation followed by full scan mass spectra was carried out to record retention time, molecular weight (m/z), and MS/MS of all detectable ions presented in the samples. Metabolites were identified by automated comparison of the ion features in the experimental samples with a reference library of chemical standard entries that included retention time, molecular weight (m/z), preferred adducts, and in-source fragments as well as their associated MS/MS spectra. This library allowed the rapid identification of metabolites in the experiment with high confidence.

### Statistical Analysis

Statistical analysis (a parametric independent t test, Mann-Whitney test, and the Wilcoxon sign rank test) was performed with STATA. Variables with a P value of less than 0.05 were said to be statistically significant. All variables remained significant after multiple testing. Custom R scripts employing the vegan [40] and ade4 [41] packages were used for the principal component analysis (PCA) and between-class correspondence analysis (BCA), respectively. Partial least squares discriminant analysis (PLS-DA), implemented by the MetaboAnalyst 2.0 software [42], was applied to the metabolite dataset from the two subject classes (stable and exacerbated). The data were mean-centred and range scaled and differences between the two classes determined by PLS-DA were validated by leave-one out cross-validation. The statistical significance of the resulting separation was determined via a permutation test, and the quality of the resulting model determined via the R^2^ and Q^2^ parameters. The MetaboAnalyst 2.0 software was also used to generate Variable Importance on Projection (VIP) scores [43], [44] which may identify the best variables for discriminating between subject classes.

## Supporting Information

Figure S1 **Relative abundances of bacterial orders identified as operational taxonomic units (OTUs) from sequence reads using 16S rRNA gene-based 454 sequencing among sputum samples taken from stable CF patients.** Percentage of sequences from total DNA (A) or total transcribed RNA (B) taken each sputum sample most closely related to 16S rRNA gene sequences from particular phylogenetic subgroups of bacteria are shown. Total bacterial density in each sputum sample examined is indicated as 16S rRNA copies/ml sputum quantitative PCR.
(PDF)

Figure S2 **Shannon–Weaver diversity indices are extremely close for the bacterial communities identified by examining DNA or RNA from stable patients and DNA or RNA from exacerbated patients.** Values in the same column followed by different letters are significantly different (p-values from paired t-test: 0.89 & 0.19, respectively).
(PDF)

Figure S3 **Identification of anaerobic bacterial populations among sputum samples from stable CF patients.** The coloured segments of each bar represent the proportion of reads mapping to different anaerobic bacterial orders. Percentage of sequences from total DNA (A) or total transcribed RNA (B) taken from sputum sample most closely related to 16S rRNA gene sequences from particular phylogenetic subgroups of bacteria.
(PDF)

Figure S4 **Prevalence of emerging CF bacterial pathogens among sputum samples from stable CF patients.** The coloured segments of each bar represent the proportion of reads mapping to different anaerobic bacterial orders. Percentage of sequences from total DNA (A) or total transcribed RNA (B) taken from sputum sample most closely related to 16S rRNA gene sequences from particular phylogenetic subgroups of bacteria.
(PDF)

Figure S5 **Plot of the Variable Importance on Projection (VIP) scores for the top 15 ranked variables (metabolites in this case).** The VIP score summarises a variables contribution to the model and is a measure of its power to discriminate between sample classes. Scores<0.80 are considered ‘small’ (43). Blue circles represent stable samples while the red diamonds represent samples from patients suffering exacerbation.
(PDF)

Table S1 **Clinical data associated with CF and non-CF patients included in this study.**
(PDF)

Table S2 **Sampling depth and biodiversity found by bar-coded 454 sequencing of sputum samples from the three patient cohorts.**
(PDF)

Table S3 **Microorganisms recovered from sputum taken from three patient cohorts included in the study.**
(PDF)

Table S4 **Concentrations of metabolites identified in CF sputum taken from 26 stable CF patients.** Data are given as the average values measured in triplicate (metabolite concentrations are described as µM unless otherwise stated and means ± standard deviations are reported).
(PDF)

Table S5 **Concentrations of metabolites identified in CF sputum taken from 26 CF patients presenting with exacerbation.** Data are given as the average values measured in triplicate (metabolite concentrations are described as µM unless otherwise stated and means ± standard deviations are reported).
(PDF)
