# Supplementary figures and images for: Microbiota and Metabolite Profiling Reveal Specific Alterations in Bacterial Community Structure and Environment in the Cystic Fibrosis Airway during Exacerbation
Source: PLoS One. 2013 Dec 17;8(12):e82432. doi: 10.1371/journal.pone.0082432 (PMC3866110; doi:10.1371/journal.pone.0082432)

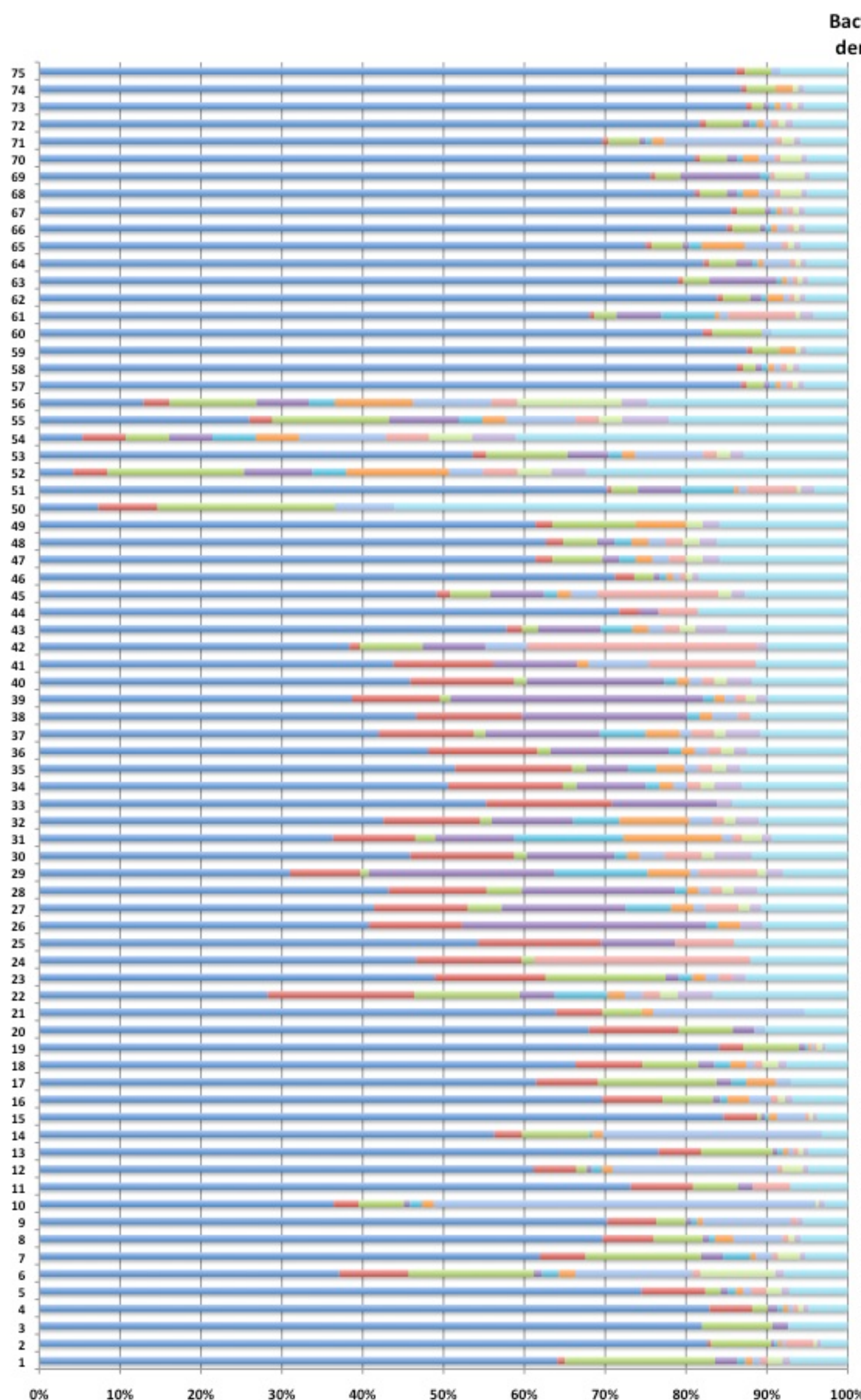

% Relative abundance of OTUs from DNA

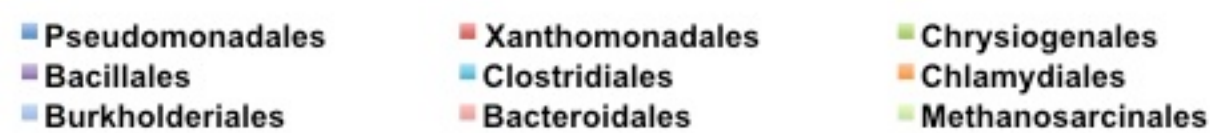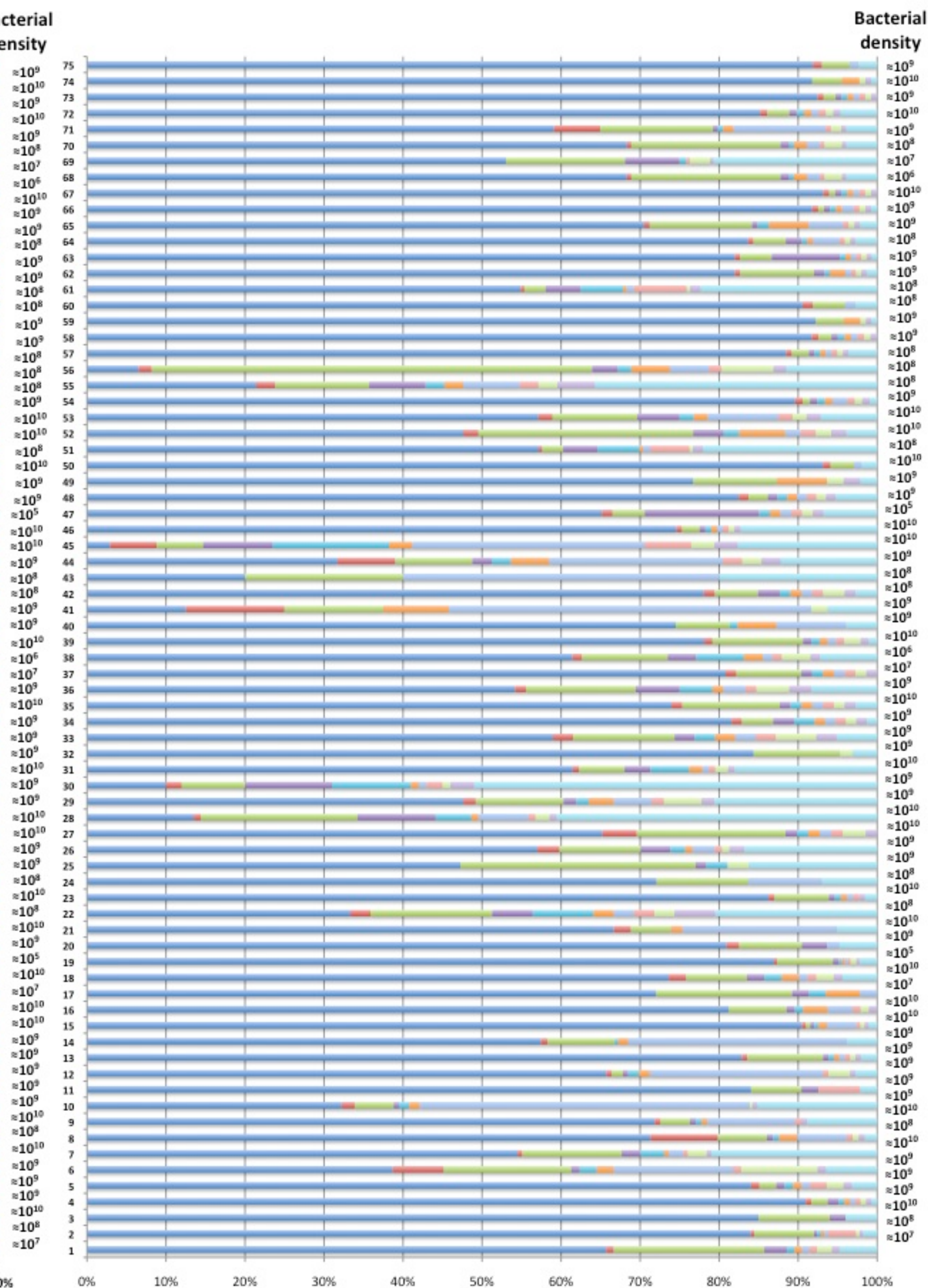

% Relative abundance of OTUs from RNA

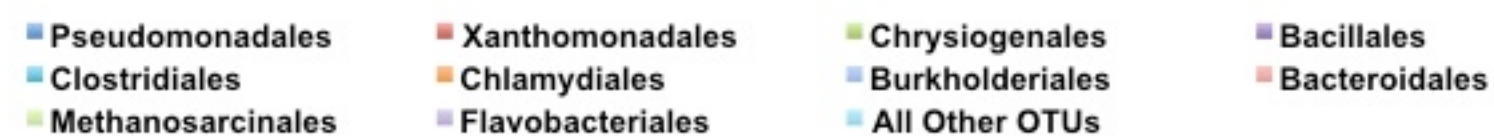

Supplement: Figure S1 — Relative abundances of bacterial orders identified as operational taxonomic units (OTUs) from sequence reads using 16S rRNA gene-based 454 sequencing among sputum samples taken from stable CF patients. Percentage of sequences from total DNA (A) or total transcribed RNA (B) taken each sputum sample most closely related to 16S rRNA gene sequences from particular phylogenetic subgroups of bacteria are shown. Total bacterial density in each sputum sample examined is indicated as 16S rRNA copies/ml sputum quantitative PCR. (PDF) [file pone.0082432.s001.pdf]

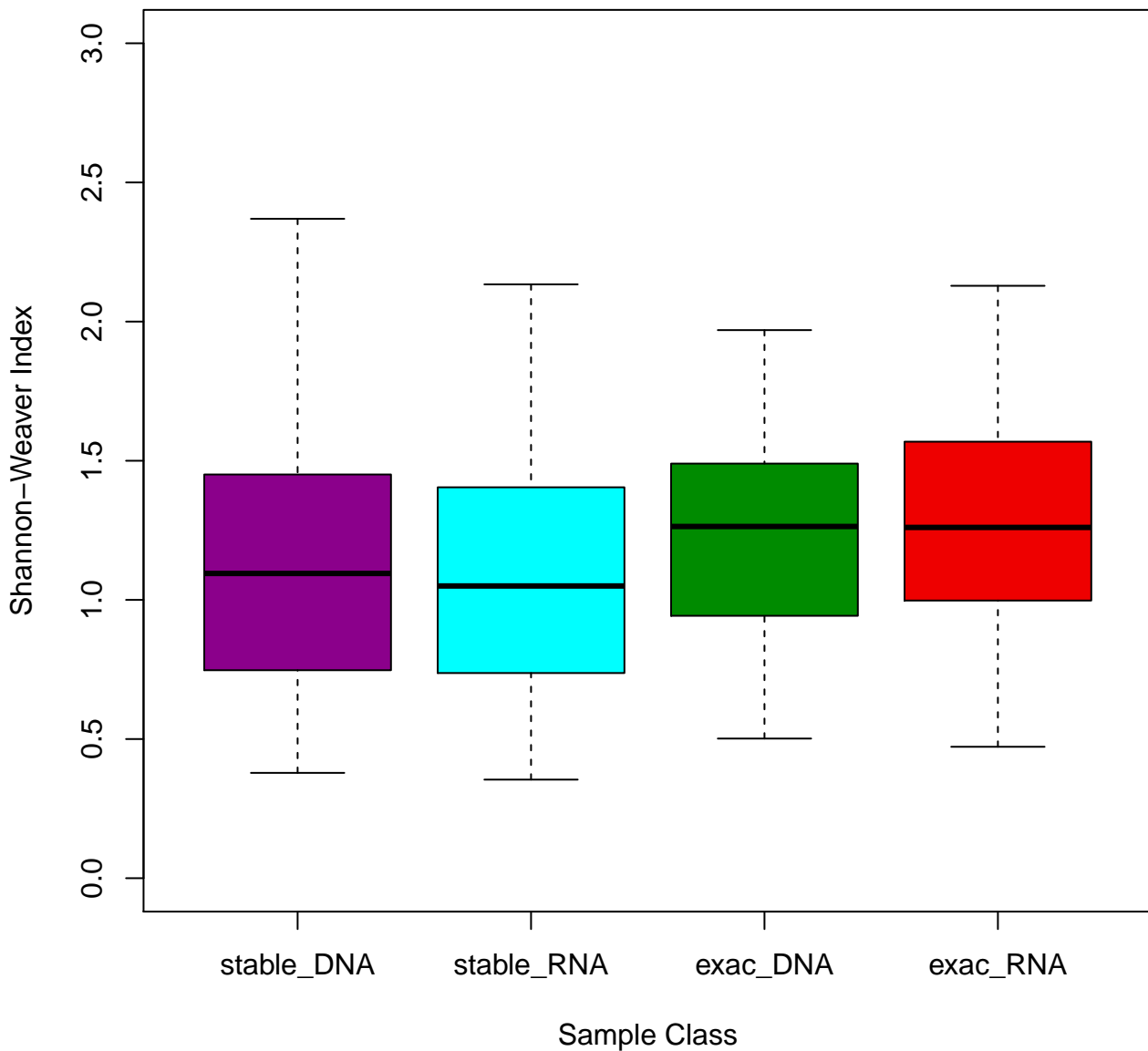

Supplement: Figure S2 — Shannon–Weaver diversity indices are extremely close for the bacterial communities identified by examining DNA or RNA from stable patients and DNA or RNA from exacerbated patients. Values in the same column followed by different letters are significantly different (p-values from paired t-test: 0.89 & 0.19, respectively). (PDF) [file pone.0082432.s002.pdf]

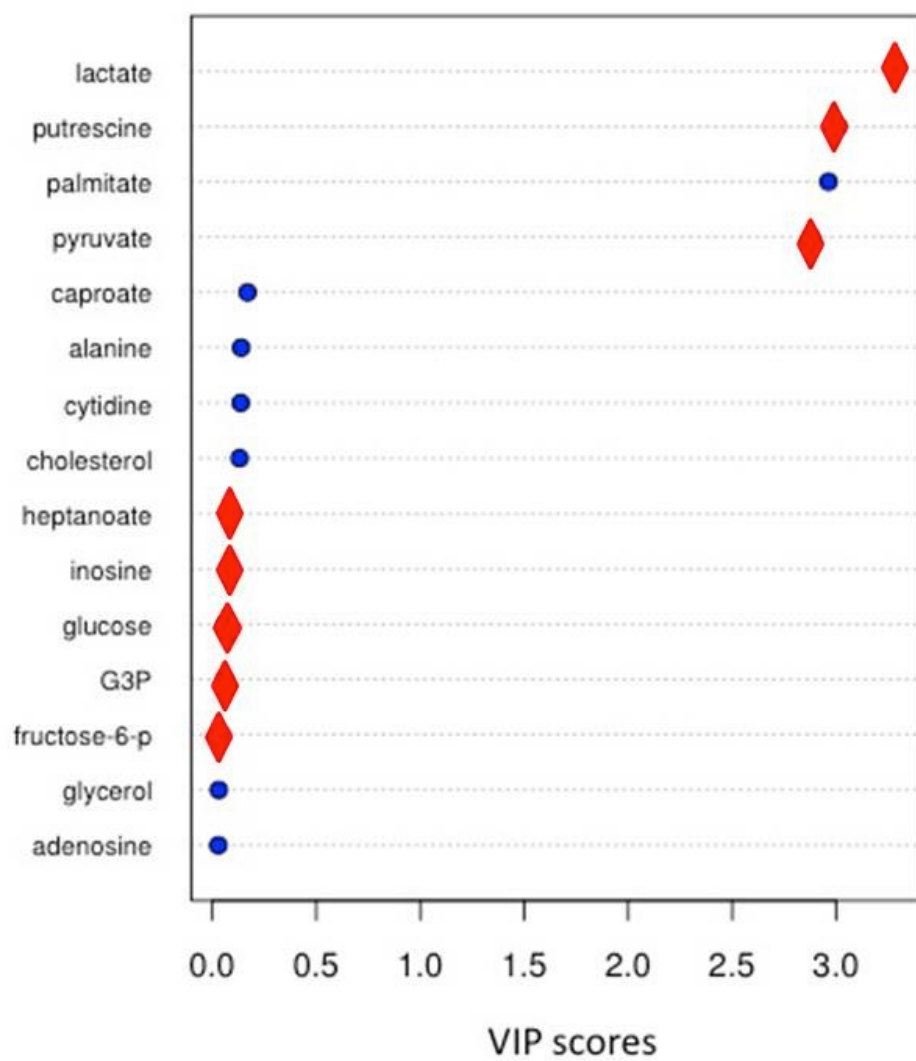

Supplement: Figure S5 — Plot of the Variable Importance on Projection (VIP) scores for the top 15 ranked variables (metabolites in this case). The VIP score summarises a variables contribution to the model and is a measure of its power to discriminate between sample classes. Scores<0.80 are considered ‘small’ (43). Blue circles represent stable samples while the red diamonds represent samples from patients suffering exacerbation. (PDF) [file pone.0082432.s005.pdf]
