# Supplementary material for: Microbiota and Metabolite Profiling Reveal Specific Alterations in Bacterial Community Structure and Environment in the Cystic Fibrosis Airway during Exacerbation
Source: PLoS One. 2013 Dec 17;8(12):e82432. doi: 10.1371/journal.pone.0082432 (PMC3866110; doi:10.1371/journal.pone.0082432)
Supplement: Figure S3 — Identification of anaerobic bacterial populations among sputum samples from stable CF patients. The coloured segments of each bar represent the proportion of reads mapping to different anaerobic bacterial orders. Percentage of sequences from total DNA (A) or total transcribed RNA (B) taken from sputum sample most closely related to 16S rRNA gene sequences from particular phylogenetic subgroups of bacteria. (PDF) [file pone.0082432.s003.pdf]

A.

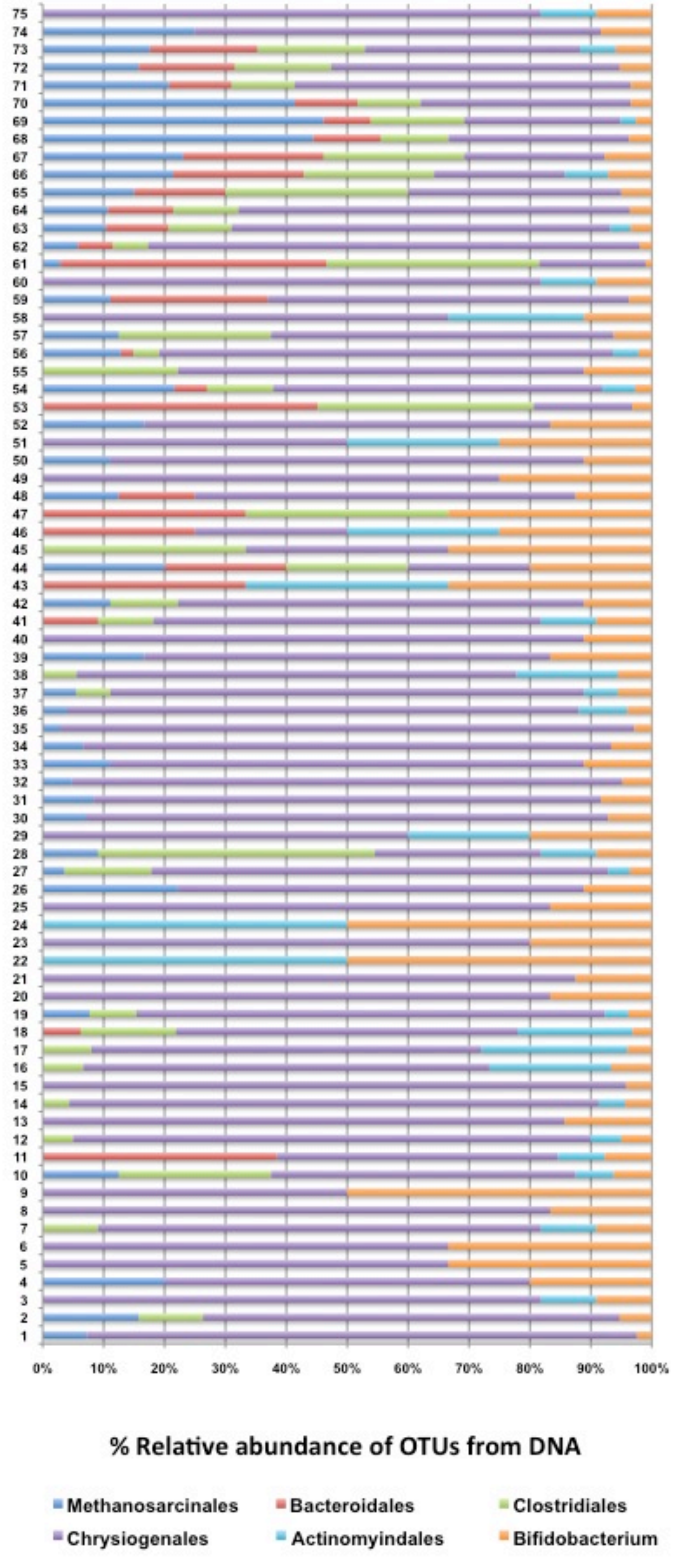

0%
10%
20%
30%
40%
50%
60%
70%
80%
90%
100%
% Relative abundance of OTUs from DNA
Methanosarcinales
Bacteroidales
Clostridiales
Chrysiogenales
Actinomyindales
Bifidobacterium

B.

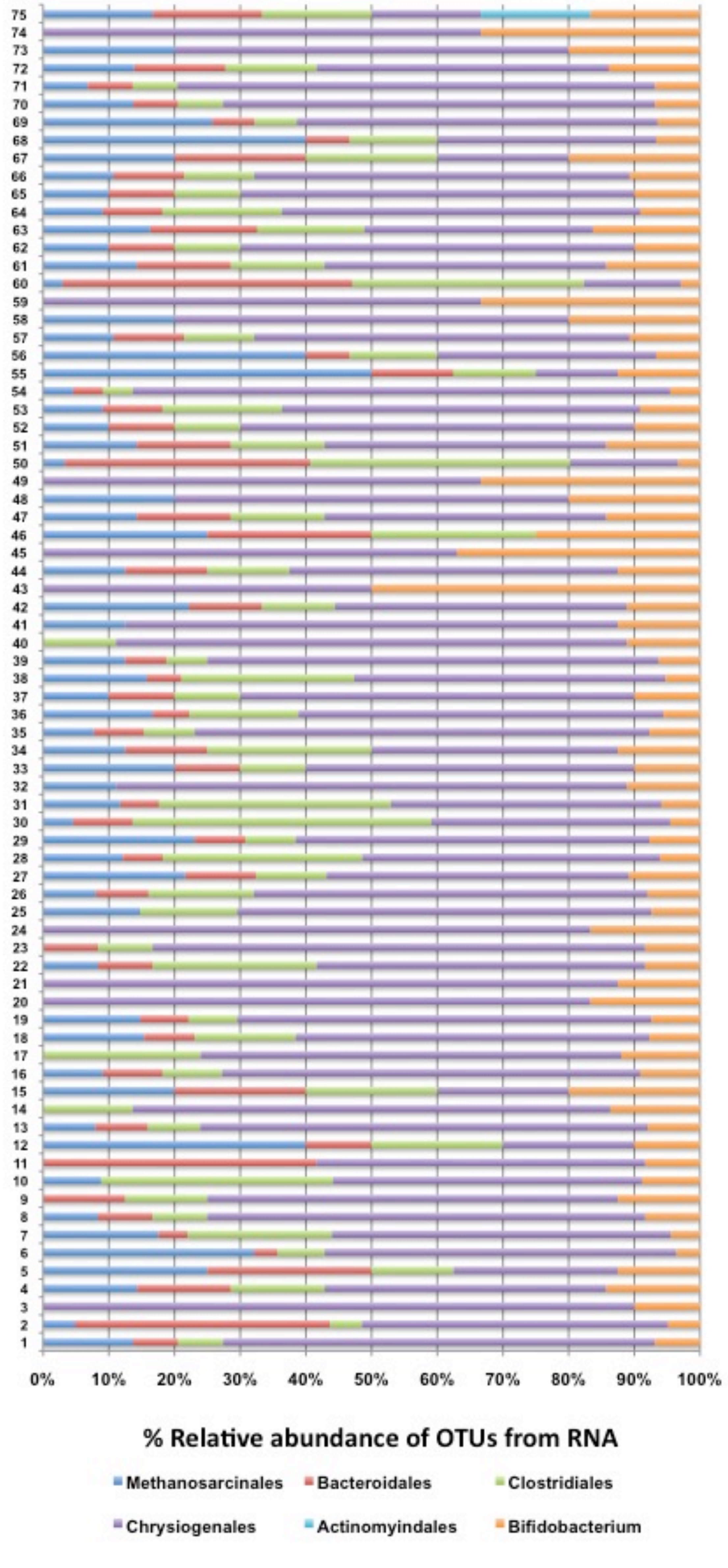

0%
10%
20%
30%
40%
50%
60%
70%
80%
90%
100%
% Relative abundance of OTUs from RNA
Methanosarcinales
Bacteroidales
Clostridiales
Chrysiogenales
Actinomyindales
Bifidobacterium
