# Supplementary material for: Microbiota and Metabolite Profiling Reveal Specific Alterations in Bacterial Community Structure and Environment in the Cystic Fibrosis Airway during Exacerbation
Source: PLoS One. 2013 Dec 17;8(12):e82432. doi: 10.1371/journal.pone.0082432 (PMC3866110; doi:10.1371/journal.pone.0082432)
Supplement: Table S1 — Clinical data associated with CF and non-CF patients included in this study. (PDF) [file pone.0082432.s006.pdf]

Table S1. **Clinical data and treatment from CF and non-CF patients included in the study.**

			Clinical data patients			Treatment
**Patient no.**	**Age (year)**	**Sex**	**Known CFTR mutation[a]**	**BMI[b]**	**FEV1[c]**	**AZ[d]**
1	20	F	ΔF508	-	76	Y
2	34	M	ΔF508	29.1	53	Y
8	27	M	ΔF508	21.9	51	Y
10	53	F	ΔF508	21	44	Y
11	24	F	ΔF508	22.3	65	N
12	38	F	ΔF508	17.1	27	Y
13	29	F	ΔF508	16.5	64	Y
16	22	F	F50Y8	23.9	100	Y
17	21	F	ΔF508	20.1	98	N
19	19	F	ΔF508	-	29	
20	23	F	ΔF508	17.3	25	Y
21	31	F	ΔF508	20.8	48	Y
26	37	M	ΔF508	24.8	32	Y
27	20	M	ΔF508	-	77	Y
32	32	M	ΔF508	18.8	40	Y
33	27	M	ΔF508	21.9	60	Y
34	27	M	G551D	2.06	50	Y
35	31	M	ΔF508	20	20	Y
40	26	F	ΔF508	21.7	62	Y
43	24	F	ΔF508	34.4	93	Y
45	27	M	ΔF508	21.9	69	Y
46	28	M	ΔF508	27.1	95	N
47	28	M	ΔF508	27.1	95	N
48	26	F	ΔF508	24.5	63	Y
54	26	F	ΔF508	-	27	Y
55	20	F	ΔF508	18.5	24	N
57	26	F	ΔF508	23.1	25	Y
58	22	M	ΔF508	-	38	
59	37	F	ΔF508	18	24	Y

59	37	F	ΔF508	18	60	Y
62	19	M	ΔF508	-	-	Y
63	27	M	ΔF508	24.3	32	Y
70	19	F	ΔF508	20.2	70	Y
72	30	M	ΔF508	25.2	69	Y
74	23	M	ΔF508	23.4	87	N
75	23	M	ΔF508	23.4	87	N
76	39	M	ΔF508	19	33	-
78	24	F	ΔF508	19	49	Y
79	38	F	G551D	20.4	82	Y
80	23	F	ΔF508	18	41	Y
80	23	F	ΔF508	18	41	Y
83	43	F	ΔF508	17.9	24	N
84	29	F	ΔF508	22.5	47	Y
85	32	M	ΔF508	20.7	54	N
89	25	M	ΔF508	18	63	Y
92	27	M	ΔF508	19.7	67	Y
93	25	M	G551D	24	84	N
97	23	F	ΔF508	19	23	Y
99	26	M	ΔF508	19.1	27	Y
27		F	ΔF508	23.1	61	Y
101	37	M	ΔF508	23	70	Y
102	28	F	ΔF508	19.5	54	Y
103	29	F	ΔF508	20.5	70	-
104	31	M	ΔF508	21	85	N
105	30	M	ΔF508	21	44	Y
106	26	F	ΔF508	21.4	84	Y
107	21	M	ΔF508	22	97	Y
110	40	M	ΔF508	23	37	Y
111	28	F	ΔF508	19.5	62	N
113	26	M	ΔF508	24.3	55	Y
119	30	M	ΔF508	18.4	61	Y
121	20	F	ΔF508	20.4	64	-
123	25	F	F5Y08	18.5	100	Y
124	28	M	R560T	26.4	48	Y

126	22	F	ΔF508	15.8	30	N
128	28	M	ΔF508	24	29	Y
129	26	M	ΔF508	20	48	Y
129	26	M	ΔF508	20	48	Y
130	30	F	ΔF508	21.4	84	N
131	27	M	Non-CF	23.8	69	N
132	38	M	Non-CF	-	36	Y
133	25	F	Non-CF	-	77	-
134	57	F	Non-CF	-	55	-
135	53	F	ΔF508	21	44	Y
136	30	M	Non-CF	19.4	49	Y

a. Type of mutation detected in the CFTR, cystic fibrosis transmembrane conductance regulator;
b. BMI, body mass index;
c. FEV1, forced expiratory volume;
d. Patient is undergoing azithromycin (AZ) treatment where “Y” corresponds to Yes and N corresponds to “No”.
