# Supplementary material for: Microbiota and Metabolite Profiling Reveal Specific Alterations in Bacterial Community Structure and Environment in the Cystic Fibrosis Airway during Exacerbation
Source: PLoS One. 2013 Dec 17;8(12):e82432. doi: 10.1371/journal.pone.0082432 (PMC3866110; doi:10.1371/journal.pone.0082432)
Supplement: Table S2 — Sampling depth and biodiversity found by bar-coded 454 sequencing of sputum samples from the three patient cohorts. (PDF) [file pone.0082432.s007.pdf]

Table S2. Sampling depth and biodiversity found by barcoded 454 sequencing of sputum samples from "stable", "exacerbated" and "control" patient cohorts

Sampling depth	Average from stable CF samples	Average from excerbated CF samples	Average from non-CF samples
**Total sequences**	60,500	65,000	71,000
**High quality pyrosequences**	49,000	50,000	51,000
**Unique sequences (100% ID)**	1,600	1,550	1,700
**OTU (98% ID)**	400	450	450
**Genera**	25-35	15-20	20-28
