# Supplementary material for: Microbiota and Metabolite Profiling Reveal Specific Alterations in Bacterial Community Structure and Environment in the Cystic Fibrosis Airway during Exacerbation
Source: PLoS One. 2013 Dec 17;8(12):e82432. doi: 10.1371/journal.pone.0082432 (PMC3866110; doi:10.1371/journal.pone.0082432)
Supplement: Table S3 — Microorganisms recovered from sputum taken from three patient cohorts included in the study. (PDF) [file pone.0082432.s008.pdf]

Table S3. Microorganisms recovered from sputum taken from three patient cohorts included in the study.

Sample designation[a]	Age (years)	Sex	Known CFTR mutation[b]	BMI[c]	FEV1[d]	Treatment with AZ[e]	Culture Data (Day of sample)[f]
E9	31	M	ΔF508	20	20	Y	BCC
E11	19	F	ΔF508	-	29	N	NA
E22	30	M	ΔF508	21	44	Y	PA
E23	26	F	ΔF508	-	27	Y	PA
E35	23	F	ΔF508	19	23	Y	PA
E37	26	F	ΔF508	23.1	25	Y	PA, CAN
E39	28	F	ΔF508	19.5	54	Y	NA
E40	19	F	ΔF508	-	29	N	PA
E44	27	F	ΔF508	23.1	61	Y	PA
E61	26	F	ΔF508	23.1	25	Y	NRF
E62	26	F	ΔF508	-	27	Y	PA
E72	32	M	ΔF508	20.7	54	N	MSSA, PA
E74	25	M	G551D	24	84	N	NA
E76	34	M	ΔF508	29.1	53	Y	PA, MRSA
E77	27	M	G551D	2.06	50	Y	MSSA
E89	27	M	ΔF508	24.3	32	Y	MSSA, CAN, ASP
E101	25	M	ΔF508	18	63	Y	PA
E104	37	F	ΔF508	18	24	Y	PA, CAN
E105	30	M	ΔF508	18.4	61	Y	PA
E108	27	F	ΔF508	18.4	52	Y	NA
E109	26	F	ΔF508	21.4	84	Y	PA, MSSA
E113	29	F	ΔF508	20.5	70	N	PA,
E116	19	M	ΔF508	-	-	Y	NA
E118	25	F	F5Y08	18.5	100	Y	PA
E123	22	F	F50Y8	23.9	100	Y	MRSA
E124	28	M	ΔF508	24	29	Y	NA
S1	28	F	G551D	22.1	41	Y	CAN, MRSA
S2	20	M	ΔF508	-	77	Y	MSSA, SA
S3	28	M	R560T	26.4	48	Y	PA
S4	27	M	ΔF508	19.7	67	Y	PA
S5	53	F	ΔF508	21	44	Y	NPC
S6	23	M	ΔF508	23.4	87	N	MSSA
S7	24	F	ΔF508	34.4	93	Y	MRSA
S8	24	F	ΔF508	19	49	Y	PA
S10	27	F	ΔF508	18.4	61	Y	PA
S13	38	F	G551D	20.4	82	Y	PA
S14	21	M	ΔF508	22	97	Y	NA
S15	21	M	ΔF508	22	97	Y	PA
S16	27	M	ΔF508	21.9	60	Y	CAN
S17	32	M	ΔF508	18.8	40	Y	PA, MSSA

S18	43	F	ΔF508	17.9	24	N	PA
S19	20	F	ΔF508	18.5	24	N	RP
S20	23	F	ΔF508	18	41	Y	NA
S21	31	M	ΔF508	21	85	N	MSSA
S24	39	M	ΔF508	19	33	N	NA
S25	27	M	ΔF508	21.9	60	Y	CAN
S26	38	F	ΔF508	17.1	27	Y	PA
S27	32	M	ΔF508	18.8	40	Y	PA, MSSA
S28	23	F	ΔF508	17.3	25	Y	PA
S32	26	M	ΔF508	24.3	55	Y	MRSA, PA
S33	26	F	ΔF508	21.4	52	Y	PA, MSSA
S34	19	M	ΔF508	18	70	Y	PA, CAN
S38	30	F	ΔF508	21.4	84	N	PA
S41	28	M	ΔF508	27.1	95	N	MSSA, PA
S42	27	M	ΔF508	21.9	69	Y	PA, CAN
S43	26	F	ΔF508	23.1	25	Y	SM, CAN
S45	29	F	ΔF508	22.5	47	Y	PA
S46	20	F	ΔF508	-	76	Y	PA, MSSA, CAN
S47	20	M	ΔF508	-	77	Y	CAN
S48	28	F	ΔF508	19.5	54	Y	PA
S49	26	M	ΔF508	24.3	55	Y	MRSA, PA, CAN
S50	20	F	ΔF508	20.4	64	Y	PA, CAN, MC
S51	38	F	ΔF508	17.1	27	Y	PA
S54	26	M	ΔF508	20	48	Y	PA
S58	28	F	ΔF508	19.5	62	N	NA
S59	28	M	ΔF508	27.1	95	N	SA, ST
S60	37	M	ΔF508	24.8	32	Y	NRF
S63	26	F	ΔF508	24.5	63	Y	PA
S64	40	M	ΔF508	23	37	Y	PA, PF, MRSA
S65	27	M	ΔF508	21.9	69	Y	NA
S66	28	F	ΔF508	19.5	62	N	PA, COLIFORM, CAN
S68	24	F	ΔF508	22.3	65	N	MSSA, MC, HP
S69	27	M	G551D	20.6	50	Y	NA
S70	22	F	ΔF508	15.8	30	N	MSSA, PA, STP
S71	21	M	ΔF508	22	97	Y	PA
S73	21	F	ΔF508	20.1	98	N	PA
S75	25	F	ΔF508	18.5	54	Y	NA
S78	30	M	ΔF508	25.2	69	Y	PA, CAN
S80	37	M	ΔF508	23	70	Y	PA, MSSA
S82	29	F	ΔF508	16.5	64	Y	NA
S84	23	F	ΔF508	18	41	Y	PA
S85	26	F	ΔF508	21.7	62	Y	PA, MSSA

S89	34	M	ΔF508	24.3	32	Y	PA
S90	31	F	ΔF508	20.8	48	Y	PA
S95	53	F	ΔF508	21	44	Y	CAN, NRF
S102	26	M	ΔF508	20	48	Y	PA
S103	19	F	ΔF508	20.2	70	Y	NRF
S106	37	F	ΔF508	18	60	Y	PA, ASP
S107	22	M	ΔF508	-	38	N	BCC
S110	23	M	ΔF508	23.4	87	N	PA, CAN
S111	26	M	ΔF508	19.1	27	Y	PA, MSSA
S112	20	F	ΔF508	20.4	64	N	PA
S114	32	M	ΔF508	18.8	40	Y	PA, MSSA, ASP
S117	26	F	ΔF508	21.4	52	Y	PA, CAN
S119	28	M	RS60T	26.4	48	Y	NA
S120	37	M	ΔF508	24.8	32	Y	PA
S126	38	F	ΔF508	17.1	27	Y	PA
S127	37	F	ΔF508	18	60	Y	PA
S128	27	M	ΔF508	21.9	51	Y	PA, MSSA, SP
NCF91	27	M	-	23.8	69	N	NRF
NCF92	38	M	-	-	36	Y	HP
NCF93	25	F	-	-	77	N	NPC
NCF94	57	F	-	-	55	N	NPC
NCF96	30	M	-	19.4	49	Y	NPC

a. Description of sputum sample taken from patient cohorts where "E" corresponds to exacerbated cohort; "S" corresponds to stable cohort and "NCF" corresponds to non-CF cohort.
b. Type of mutation detected in the CFTR, cystic fibrosis transmembrane conductance regulator;
c. BMI, body mass index;
d. FEV1, forced expiratory volume;
e. Patient is undergoing azithromycin (AZ) treatment where "Y" corresponds to Yes and N corresponds to "No".
f. Abbreviations of microorganisms identified by culture-based methods: CAN-*Candida* species, SA-*Staphylococcus aureus*, PA-*Pseudomonas aeruginosa*, ST-*Stenotrophomonas* species, ASP-Aspergillus species, STP-*Streptococcus* species, BCC-*Burkholderia* species, SP-*Sphingomonas* species, MSSA-Methicillin Sensitive *Staphlycoccus aureus,* MRSA- Methicillin Resistant *Staphlycoccus aureus,* RP- *Ralstonia pickettii,* MC- *Moraxella catarrhalis,* SA- *Staphlycoccus aureus,* Sp - *Streptococcus pneumoniae,* Pf - *Pseudomonas fluorescens,* Hp-*Haemophilius parainfluenzae,* SM-*Stenotrophomonas maltophilia,* NPC-No pathogens cultured, NA- Not assessed.
