# Supplementary material for: Microbiota and Metabolite Profiling Reveal Specific Alterations in Bacterial Community Structure and Environment in the Cystic Fibrosis Airway during Exacerbation
Source: PLoS One. 2013 Dec 17;8(12):e82432. doi: 10.1371/journal.pone.0082432 (PMC3866110; doi:10.1371/journal.pone.0082432)
Supplement: Table S4 — Concentrations of metabolites identified in CF sputum taken from 26 stable CF patients. Data are given as the average values measured in triplicate (metabolite concentrations are described as µM unless otherwise stated and means ± standard deviations are reported). (PDF) [file pone.0082432.s009.pdf]

Table S4. Concentrations of metabolites identified in CF sputum taken from 26 stable CF patients. Data are given as the average values measured in triplicate (metabolite concentrations are described as μM unless otherwise stated and means ± standard deviations are reported).

**Metabolite**		**S1**	**S2**	**S3**	**S4**	**S5**	**S6**	**S7**	**S8**	**S9**	**S10**	**S11**	**S12**	**S13**	**S14**	**S15**	**S16**	**S17**	**S18**	**S19**	**S20**	**S21**	**S22**	**S23**	**S24**	**S25**
Amino acids	alanine	2500.0	2510.0	2555.0	2589.0	2572.0	2589.0	2549.0	2533.0	2554.0	2579.0	2577.0	2588.0	2555.0	2577.0	2599.0	2599.0	2545.0	2533.0	2500.0	2570.0	2513.0	2595.0	2955.0	2600.0	2400.0
	aspartate	64.7	63.9	64.1	65.4	64.6	66.0	65.4	64.5	64.4	65.1	64.7	65.8	64.4	64.4	65.0	66.4	65.3	65.9	63.2	66.5	62.9	57.1	73.9	70.4	53.8
	cysteine	20.3	19.9	19.6	20.3	19.6	20.2	20.3	20.0	19.8	20.1	19.7	20.4	20.0	19.8	19.6	20.3	19.8	20.8	19.0	21.1	22.1	15.5	21.0	26.2	16.6
	glycine	76.4	76.2	72.6	77.7	72.7	74.6	76.0	75.9	73.8	76.7	72.2	76.6	75.6	75.9	72.3	76.9	71.4	82.1	67.6	71.8	107.0	55.2	60.1	118.6	62.1
	glutamate	114.4	119.6	106.5	121.9	111.1	111.9	114.1	117.2	109.8	121.1	107.4	115.5	113.5	119.8	107.2	122.7	99.1	138.6	107.2	76.5	220.9	104.8	57.8	217.9	94.3
	histidine	66.0	77.5	60.3	75.7	68.5	67.7	67.2	73.7	62.5	77.8	64.1	70.2	65.4	77.2	60.2	85.4	49.3	89.2	96.7	23.9	145.5	125.1	18.3	159.4	57.1
	lysine	127.6	192.9	119.4	165.9	151.8	151.1	136.9	176.7	120.5	182.0	137.1	161.0	127.1	192.1	105.8	259.7	83.0	138.3	607.2	24.6	174.4	1026.2	17.2	418.6	124.1
	phenylalanine	26.5	64.6	28.8	43.7	40.2	44.2	31.0	58.6	26.5	53.1	34.0	50.7	26.5	72.6	16.3	125.2	24.0	9.1	986.0	5.6	6.8	2384.0	1.6	134.9	33.1
	tyrosine	49.8	49.1	49.3	50.3	49.7	50.8	50.3	49.6	49.5	50.0	49.8	50.6	49.6	49.6	50.0	51.1	50.2	50.7	48.6	51.1	48.4	43.9	56.8	54.2	41.4
	tryptophan	34.5	33.8	33.3	34.5	33.3	34.3	34.5	34.1	33.6	34.2	33.4	34.7	34.0	33.7	33.3	34.6	33.7	35.4	32.3	35.9	37.5	26.3	35.7	44.5	28.1
	arginine	91.7	91.4	87.1	93.2	87.2	89.5	91.2	91.1	88.6	92.0	86.6	92.0	90.8	91.1	86.7	92.2	85.7	98.6	81.1	86.2	128.4	66.3	72.1	142.4	74.5
	proline	150.5	157.4	140.1	160.4	146.1	147.3	150.2	154.2	144.5	159.4	141.3	152.0	149.4	157.7	141.0	161.5	130.4	182.4	141.1	100.7	290.7	137.8	76.0	286.7	124.1
	valine	1244.5	1463.0	1137.5	1428.8	1291.9	1277.0	1268.0	1389.8	1180.1	1468.9	1210.3	1324.5	1233.8	1455.8	1136.8	1612.1	931.1	1683.0	1824.9	450.9	2745.5	2361.4	345.2	3006.9	1077.5
Polyamines	putrescine	21.0	17.9	23.0	18.3	20.2	20.5	20.6	18.8	22.1	17.8	21.6	19.7	21.2	18.0	23.0	16.2	28.1	15.5	14.3	58.0	9.5	11.1	75.7	8.7	24.3
	spermine	10.0	9.8	9.9	10.1	9.9	10.2	10.1	9.9	9.9	10.0	10.0	10.1	9.9	9.9	10.0	10.2	10.0	10.1	9.7	10.2	9.7	8.8	11.4	10.8	8.3
	spermidine	8.1	8.0	7.8	8.1	7.8	8.1	8.1	8.0	7.9	8.0	7.9	8.2	8.0	7.9	7.8	8.1	7.9	8.3	7.6	8.4	8.8	6.2	8.4	10.5	6.6
Carbohydrate	fructose	81.5	81.2	77.4	82.9	77.5	79.6	81.1	81.0	78.8	81.8	77.0	81.7	80.7	81.0	77.1	82.0	76.2	87.6	72.1	76.6	114.2	58.9	64.1	126.5	66.2
	fructose-6-phosphate	90.3	94.4	84.1	96.2	87.7	88.4	90.1	92.5	86.7	95.6	84.8	91.2	89.6	94.6	84.6	96.9	78.3	109.4	84.6	60.4	174.4	82.7	45.6	172.0	74.5
	glucose	99.4	116.9	90.9	114.1	103.2	102.0	101.3	111.0	94.3	117.3	96.7	105.8	98.5	116.3	90.8	128.8	74.4	134.4	145.8	36.0	219.3	188.6	27.6	240.2	86.1
	pyruvate	11.1	11.1	10.9	10.7	10.8	10.7	10.9	11.0	10.9	10.8	10.8	10.7	10.9	10.8	10.7	10.7	10.9	11.0	11.1	10.8	11.0	10.7	9.4	10.7	11.6
	lactate	26.1	26.5	26.3	25.8	26.2	25.6	25.8	26.2	26.3	26.0	26.1	25.7	26.2	26.2	26.0	25.5	25.9	25.7	26.7	25.4	26.8	29.6	22.9	24.0	31.4
	maltose	89.6	88.4	88.8	90.6	89.4	91.4	90.6	89.3	89.1	90.1	89.6	91.2	89.2	89.2	90.0	91.9	90.4	91.2	87.5	92.0	87.2	79.0	102.3	97.5	74.5
	maltotetraose	71.0	69.7	68.6	70.9	68.5	70.7	71.0	70.1	69.3	70.4	68.8	71.5	70.0	69.4	68.5	71.1	69.4	72.9	66.6	73.9	77.2	54.1	73.4	91.6	57.9
	maltotriose	49.8	49.1	49.3	50.3	49.7	50.8	50.3	49.6	49.5	50.0	49.8	50.6	49.6	49.6	50.0	51.1	50.2	50.7	48.6	51.1	48.4	43.9	56.8	54.2	41.4
Lipid	glycerol	5.1	5.0	4.9	5.1	4.9	5.0	5.1	5.0	4.9	5.0	4.9	5.1	5.0	5.0	4.9	5.1	5.0	5.2	4.8	5.3	5.5	3.9	5.2	6.5	4.1
	glycerol 3-phosphate (G3P)	5.0	4.9	4.9	5.0	5.0	5.1	5.0	5.0	5.0	5.0	5.0	5.1	5.0	5.0	5.0	5.1	5.0	5.1	4.9	5.1	4.8	4.4	5.7	5.4	4.1
	cholesterol	4.1	4.0	3.9	4.1	3.9	4.0	4.1	4.0	4.0	4.0	3.9	4.1	4.0	4.0	3.9	4.1	4.0	4.2	3.8	4.2	4.4	3.1	4.2	5.2	3.3
	7-beta-hydroxycholesterol	32.9	32.4	32.6	33.2	32.8	33.5	33.2	32.7	32.7	33.0	32.9	33.4	32.7	32.7	33.0	33.7	33.2	33.4	32.1	33.7	32.0	29.0	37.5	35.8	27.3
	lathosterol	22.9	22.6	22.7	23.2	22.8	23.4	23.1	22.8	22.8	23.0	22.9	23.3	22.8	22.8	23.0	23.5	23.1	23.3	22.4	23.5	22.3	20.2	26.1	24.9	19.0
	oleate (18:1n9)	13.9	13.8	13.8	14.1	13.9	14.2	14.1	13.9	13.9	14.0	13.9	14.2	13.9	13.9	14.0	14.3	14.1	14.2	13.6	14.3	13.6	12.3	15.9	15.2	11.6
	palmitate (16:0)	35.0	45.0	40.0	27.0	40.0	56.0	40.0	35.0	29.0	61.0	23.0	34.0	45.0	45.0	35.0	39.0	41.0	40.0	47.0	32.0	60.0	61.0	34.0	42.0	37.0
	caproate (6:0)	117.5	119.5	118.7	119.5	117.6	116.9	117.9	119.0	118.9	119.4	117.9	118.9	120.0	120.0	117.5	116.9	115.4	118.6	116.0	119.8	136.4	120.0	110.8	133.8	133.8
	heptanoate (7:0)	7.0	6.9	6.9	7.0	7.0	7.1	7.0	6.9	6.9	7.0	7.0	7.1	6.9	6.9	7.0	7.1	7.0	7.1	6.8	7.2	6.8	6.1	8.0	7.6	5.8
Nucleotide	inosine	6.0	5.9	5.9	6.0	6.0	6.1	6.0	6.0	5.9	6.0	6.0	6.1	5.9	5.9	6.0	6.1	6.0	6.1	5.8	6.1	5.8	5.3	6.8	6.5	5.0
	adenosine	4.0	3.9	3.9	4.0	4.0	4.1	4.0	4.0	4.0	4.0	4.0	4.1	4.0	4.0	4.0	4.1	4.0	4.1	3.9	4.1	3.9	3.5	4.5	4.3	3.3
	guanosine	30.9	30.5	30.6	31.2	30.8	31.5	31.2	30.7	30.7	31.0	30.9	31.4	30.7	30.7	31.0	31.7	31.1	31.4	30.2	31.7	30.0	27.2	35.2	33.6	25.7
	cytidine	25.9	25.5	25.7	26.2	25.8	26.4	26.2	25.8	25.7	26.0	25.9	26.3	25.8	25.8	26.0	26.6	26.1	26.3	25.3	26.6	25.2	22.8	29.6	28.2	21.5
