# Supplementary material for: Microbiota and Metabolite Profiling Reveal Specific Alterations in Bacterial Community Structure and Environment in the Cystic Fibrosis Airway during Exacerbation
Source: PLoS One. 2013 Dec 17;8(12):e82432. doi: 10.1371/journal.pone.0082432 (PMC3866110; doi:10.1371/journal.pone.0082432)
Supplement: Table S5 — Concentrations of metabolites identified in CF sputum taken from 26 CF patients presenting with exacerbation. Data are given as the average values measured in triplicate (metabolite concentrations are described as µM unless otherwise stated and means ± standard deviations are reported). (PDF) [file pone.0082432.s010.pdf]

Table S5. Concentrations of metabolites identified in CF sputum taken from 26 CF patients presenting with exacerbation. Data are given as the average values measured in triplicate (metabolite concentrations are described as μM unless otherwise stated).

Metabolite		E1	E2	E3	E4	E5	E6	E7	E8	E9	E10	E11	E12	E13	E14	E15	E16	E17	E18	E19	E20	E21	E22	E23	E24	E25	E26
Amino acids	alanine	2500	2500	2510	2555	2589	2572	2589	2549	2533	2554	2579	2577	2588	2555	2577	2599	2599	2545	2533	2500	2570	2513	2595	2955	2600	2400
	aspartate	65	65	64	64	65	65	66	65	64	64	65	65	66	64	64	65	66	65	66	63	66	63	57	74	70	54
	cysteine	20	20	20	20	20	20	20	20	20	20	20	20	20	20	20	20	20	20	21	19	21	22	15	21	26	17
	glycine	75	76	76	73	78	73	75	76	76	74	77	72	77	76	76	72	77	71	82	68	72	107	55	60	119	62
	glutamate	114	114	120	106	122	111	112	114	117	110	121	107	115	114	120	107	123	99	139	107	76	221	105	58	218	94
	histidine	69	66	78	60	76	68	68	67	74	63	78	64	70	65	77	60	85	49	89	97	24	145	125	18	159	57
	lysine	150	128	193	119	166	152	151	137	177	121	182	137	161	127	192	106	260	83	138	607	25	174	1026	17	419	124
	phenylalanine	40	26	65	29	44	40	44	31	59	26	53	34	51	26	73	16	125	24	9	986	6	7	2384	2	135	33
	tyrosine	50	50	49	49	50	50	51	50	50	50	50	50	51	50	50	50	51	50	51	49	51	48	44	57	54	41
	tryptophan	34	34	34	33	34	33	34	34	34	34	34	33	35	34	34	33	35	34	35	32	36	37	26	36	45	28
	arginine	90	92	91	87	93	87	90	91	91	89	92	87	92	91	91	87	92	86	99	81	86	128	66	72	142	74
	proline	150	150	157	140	160	146	147	150	154	144	159	141	152	149	158	141	161	130	182	141	101	291	138	76	287	124
	valine	1302	1244	1463	1137	1429	1292	1277	1268	1390	1180	1469	1210	1324	1234	1456	1137	1612	931	1683	1825	451	2745	2361	345	3007	1078
Polyamines	putrescine	104	103	165	155	110	109	108	165	123	98	45	108	87	129	149	105	105	121	89	110	165	150	112	111	104	103
	spermine	10	10	10	10	10	10	10	10	10	10	10	10	10	10	10	10	10	10	10	10	10	10	9	11	11	8
	spermidine	8	8	8	8	8	8	8	8	8	8	8	8	8	8	8	8	8	8	8	8	8	9	6	8	10	7
Carbohydrate	fructose	80	81	81	77	83	78	80	81	81	79	82	77	82	81	81	77	82	76	88	72	80	114	59	64	127	66
	fructose-6-phosphate	90	90	94	84	96	88	88	90	93	87	96	85	91	90	95	85	97	78	109	85	90	174	83	46	172	74
	glucose	104	99	117	91	114	103	102	101	111	94	117	97	106	99	116	91	129	74	134	146	104	219	189	28	240	86
	pyruvate	250	255	310	410	267	255	245	295	395	235	250	239	240	396	410	235	250	241	244	264	355	500	235	264	335	254
	lactate	383	375	520	510	283	395	411	341	255	428	402	421	419	254	595	397	373	387	382	365	510	580	334	397	311	410
	maltose	90	90	88	89	91	89	91	91	89	89	90	90	91	89	89	90	92	90	90	88	92	87	79	102	98	74
	maltotetraose	70	71	70	69	71	68	71	71	70	69	70	69	72	70	69	69	71	69	70	67	74	77	54	73	92	58
	maltotriose	50	50	49	49	50	50	51	50	50	50	50	50	51	50	50	50	51	50	50	49	51	48	44	57	54	41
Lipid	glycerol	5	5	5	5	5	5	5	5	5	5	5	5	5	5	5	5	5	5	5	5	5	6	4	5	7	4
	glycerol 3-phosphate (G3P)	5	5	5	5	5	5	5	5	5	5	5	5	5	5	5	5	5	5	5	5	5	5	4	6	5	4
	cholesterol	4	4	4	4	4	4	4	4	4	4	4	4	4	4	4	4	4	4	4	4	4	4	3	4	5	3
	7-beta-hydroxycholesterol	33	33	32	33	33	33	34	33	33	33	33	33	33	33	33	33	34	33	33	32	34	32	29	38	36	27
	lathosterol	23	23	23	23	23	23	23	23	23	23	23	23	23	23	23	23	23	23	23	22	24	22	20	26	25	19
	oleate (18:1n9)	14	14	14	14	14	14	14	14	14	14	14	14	14	14	14	14	14	14	14	14	14	14	12	16	15	12
	palmitate (16:0)	5	4	3	5	9	6	4	11	2	4	2	9	11	7	8	4	3	4	5	0	1	7	3	4	5	5
	caproate (6:0)	3	117	115	119	116	120	136	120	111	134	134	134	119	118	117	118	119	119	118	119	118	119	120	120	117	117
	heptanoate (7:0)	7	7	7	7	7	7	7	6	8	8	6	7	7	7	7	7	7	7	7	7	7	7	7	7	7	7
Nucleotide	inosine	6	6	6	6	6	6	6	5	7	7	5	6	6	6	6	6	6	6	6	6	6	6	6	6	6	6
	adenosine	4	4	4	4	4	4	4	4	5	4	3	4	4	4	4	4	4	4	4	4	4	4	4	4	4	4
	guanosine	31	32	31	31	30	32	30	27	35	34	26	31	31	31	31	31	31	31	31	31	31	31	31	31	31	31
	cytidine	10	27	26	26	25	27	25	23	30	28	22	26	26	26	26	26	26	26	26	26	26	26	26	26	26	26
